# Supplementary material for: Investigating latent mean differences in achievement emotions among Chinese secondary EFL learners: A gender and grade perspective
Source: PLoS One. 2024 May 31;19(5):e0303965. doi: 10.1371/journal.pone.0303965 (PMC11142686; doi:10.1371/journal.pone.0303965)
Supplement: Attachment — Submitted filename: Response to reviewers.docx [file pone.0303965.s003.docx]

Dear Editor and Reviewers,
Thank you for your encouragement and giving us the opportunity to continue revising. We have re-read the submission format requirements and made modifications based on your editing suggestions. We received 5 editing suggestions in your previous email, and we have made the necessary changes and responded to them. We look forward to responding and revising seriously and responsibly. However, if there is any misunderstanding due to communication, please let us know directly. We will be grateful for your feedback and make the necessary improvements to enhance the quality of the manuscript.

*1. Please ensure that your manuscript meets PLOS ONE's style requirements, including those for file naming. The PLOS ONE style templates can be found at* [*https://journals.plos.org/plosone/s/file?id=wjVg/PLOSOne_formatting_sample_main_body.pdf*](https://journals.plos.org/plosone/s/file?id=wjVg/PLOSOne_formatting_sample_main_body.pdf) *and*[*https://journals.plos.org/plosone/s/file?id=ba62/PLOSOne_formatting_sample_title_authors_affiliations.pdf*](https://journals.plos.org/plosone/s/file?id=ba62/PLOSOne_formatting_sample_title_authors_affiliations.pdf)

Responses: Thank you for your valuable comments and suggestions. We re-read the formatting requirements outlined by PLOS One and made appropriate adjustments to our manuscript to align with those standards. Specifically, Further modifications have been made to the format of the title page, page numbers, line spacing, table format, ensuring figures do not appear in the main text, and the placement of supporting information.

*2.* *Note from Emily Chenette, Editor in Chief of PLOS ONE, and Iain Hrynaszkiewicz, Director of Open Research Solutions at PLOS: Did you know that depositing data in a repository is associated with up to a 25% citation advantage (https://doi.org/10.1371/journal.pone.0230416)? If you’ve not already done so, consider depositing your raw data in a repository to ensure your work is read, appreciated and cited by the largest possible audience. You’ll also earn an Accessible Data icon on your published paper if you deposit your data in any participating repository (*[*https://plos.org/open-science/open-data/#accessible-data*](https://plos.org/open-science/open-data/#accessible-data)*).*

Responses: We fully endorse the concept of sharing data to advance scientific research. In addition to uploading supplementary files to the submission system, following your suggestion, we have also deposited the raw data in the Zenodo repository. You can access the data at https://doi.org/[10.5281/zenodo.11095878](https://zenodo.org/doi/10.5281/zenodo.11095878). Through Zenodo, we make our data available to the public without any restrictions.

*3. Thank you for stating the following financial disclosure: "Yajun Wu was awarded the fund by the 2023 Guangdong Philosophy and Social Science Foundation Special Projects (Grant number: GD23WZXY02-02)." Please state what role the funders took in the study.  If the funders had no role, please state: "**The funders had no role in study design, data collection and analysis, decision to publish, or preparation of the manuscript. If this statement is not correct you must amend it as needed. Please include this amended Role of Funder statement in your cover letter; we will change the online submission form on your behalf.*

Responses: The corresponding author, Yajun Wu, received financial support for this study. On the one hand, the funding was provided by an organization rather than an individual, primarily for expenses such as printing paper questionnaires and covering travel costs for researchers. The funding organization did not participate in the study design, data collection and analysis, decision to publish, or manuscript preparation. On the other hand, the funding agency requires the disclosure of this funding information. All aspects of the paper’s design and writing are disclosed in the section of “Author contributions”.

*4. Please include your full ethics statement in the ‘Methods’ section of your manuscript file. In your statement, please include the full name of the IRB or ethics committee who approved or waived your study, as well as whether or not you obtained informed written or verbal consent. If consent was waived for your study, please include this information in your statement as well.*

Responses: Thank you for your valuable suggestions. Both ethics approval and written consent were obtained for the present study. According to your suggestions, the ethics statement was included in the section of “Methods”.

*5.* *When completing the data availability statement of the submission form, you indicated that you will make your data available on acceptance. We strongly recommend all authors decide on a data sharing plan before acceptance, as the process can be lengthy and hold up publication timelines. Please note that, though access restrictions are acceptable now, your entire data will need to be made freely accessible if your manuscript is accepted for publication. This policy applies to all data except where public deposition would breach compliance with the protocol approved by your research ethics board. If you are unable to adhere to our open data policy, please kindly revise your statement to explain your reasoning and we will seek the editor's input on an exemption. Please be assured that, once you have provided your new statement, the assessment of your exemption will not hold up the peer review process.*

Responses: Considering your suggestion and the benefits of data sharing for academia and readers, I and all the co-authors of this study decided to share all the data used in this study through the Zenodo platform ([https://doi.org/[10.5281/zenodo.11095878](https://zenodo.org/doi/10.5281/zenodo.11095878)](https://doi.org/10.5061/dryad.3ffbg79rw)). We will also adhere to data sharing in the future to better serve readers and academia. Thank you for your valuable comments and suggestions.
